# Comparing two dry needling interventions for plantar heel pain: a protocol for a randomized controlled trial

**DOI:** 10.1186/s13018-019-1066-4

**Published:** 2019-01-25

**Authors:** Zaid Al-Boloushi, Eva María Gómez-Trullén, Pablo Bellosta-López, María Pilar López-Royo, Daniel Fernández, Pablo Herrero

**Affiliations:** 1 Department of Physical Medicine and Rehabilitation and Nursing, Health Sciences Faculty, University of Zaragoza, C/ Domingo Miral s/n, 50009 Zaragoza, Spain; 2 iPhysio Research Group, Universidad San Jorge, Campus Universitario, Autov. A23 km 299, 50830. Villanueva de Gállego, Zaragoza, Spain; 3 Ministry of Health, State of Kuwait, Jamal Abdulnasser Street, Al Solaibeykhat Area Safat, 13001 Kuwait City, Kuwait

**Keywords:** Plantar heel pain, Myofascial trigger points, Dry needling, Percutaneous needle electrolysis, Self-stretching protocol, Cost-effectiveness

## Abstract

**Background:**

Both manual therapy techniques and dry needling have shown to be effective treatment options for the treatment of plantar heel pain; however, in recent years, other techniques based on dry needling (DN), such as percutaneous needle electrolysis (PNE), have also emerged. Currently, PNE is being used in clinical practice to manage myofascial trigger points, despite the lack of studies comparing the effects of this technique over dry needling. Therefore, the aim of this randomized controlled study is to compare the effectiveness of DN versus PNE for improving the level of pain experienced by patients suffering from plantar heel pain provoked by myofascial trigger points.

**Methods:**

A randomized controlled trial will be conducted with blinded participants and outcome assessors. A sample of 94 patients with a medical diagnosis of plantar heel pain will be recruited and divided into two treatment groups. Eligible participants will be randomly allocated to either (a) treatment group with DN and a self-stretching home program or (b) treatment group with PNE and a self-stretching home program. Each group will receive one treatment session per week over a period of 4 weeks. The primary outcome measure will be the pain subscale of the Foot Health Status Questionnaire. The secondary outcome measures will be a visual analogue scale for pain (average and highest level of pain experienced during the previous 48 h; level of pain immediately after the treatment session) and health-related quality of life (assessed using the EuroQoL-5 dimensions). Cost-effectiveness data will be extracted based on the EuroQoL-5 dimensions. Follow-up measurements will take place at baseline and at 4, 8, 12, 26, and 52 weeks.

**Discussion:**

The justification for this trial is the need to improve current understanding regarding the effectiveness of treatments targeting the rehabilitation of plantar heel pain. This study will be the first randomized controlled trial to directly compare the effectiveness of DN and PNE combined with a specific stretching program for the treatment of plantar heel pain provoked by myofascial trigger points.

**Trial registration:**

Clinical Trials NCT03236779. Registered at clinicaltrials.gov 2 August 2017.

## Background

Plantar heel pain (PHP) is one of the main sources of pain in the foot, causing soreness or tenderness in the sole of the foot, under the heel, and which sometimes extends into the medial arch [1, 2]. This condition affects both athletic and sedentary individuals and does not seem to be influenced by gender [2]. The incidence and prevalence of plantar heel pain is uncertain; however, it is estimated that over the course of a lifetime, 10% of the population may suffer this condition [3, 4]. Furthermore, results from a high-quality epidemiological study in the USA from the 1990s found that approximately one million patient visits to physicians per year were due to PHP [5], with an associated annual cost of around $300 million [6].

Plantar heel pain may include different sources of pain, involving various diagnoses, such as myofascial pain syndrome, plantar fasciitis, or heel spur, among others [7]. The diagnosis is usually made based on the patient’s history and physical examination, including pain during the first steps in the morning or after prolonged rest, as well as pain during prolonged standing or walking [3, 4, 6]; more in-depth examinations are used only to rule out other disorders causing inferior heel pain, such as tumors, infections, and neuropathic pain (including tarsal tunnel syndrome) [8, 9]. The proper identification of the main cause of pain can be difficult as, usually, this may be multifactorial [10]. Current heel pain guidelines identify risk factors that include limited ankle dorsiflexion ROM, high body mass index (BMI) in nonathletic individuals, running, and work-related weight-bearing activities [3, 4].

There is a lack of consensus regarding the ideal management approach for PHP [11–13]. Clinical practice guidelines support the use of conservative treatment, such as joint and soft tissue mobilization or self-stretching home programs (SSHP) [3, 4]. In particular, SSHP has shown to be effective for addressing PHP [3, 10, 14], while recent randomized clinical trials (RCTs) have shown that there is an additional effect reducing the severity of pain when SSHP is combined with ischemic compression [15] and with dry needling (DN) [16].

Despite its prevalence, the etiology of PHP is not well understood [3, 4]. Although PHP may be provoked by a tendinous injury affecting the plantar fascia, it is well known that the presence of myofascial trigger points (MTrPs) within the plantar and lower leg musculature may play an important role in people with PHP [17], and recent studies have based their hypothesis on this assumption [15, 16, 18, 19]. Some of these have demonstrated the effectiveness of manual therapy techniques (i.e., ischemic compression) [15, 19] while others have also demonstrated the effectiveness of DN [16, 18].

Physical therapy approaches continue to evolve and include the combination of DN and electrolysis, known as percutaneous needle electrolysis (PNE), with promising results for the treatment of tendon pathologies [20–22]. The PNE technique is a minimally invasive treatment that consists of the application of a galvanic electrolytic current that causes a controlled local inflammatory process in the target tissue. This promotes phagocytosis and the subsequent regeneration of the affected tissue [20, 21]. Nowadays, PNE is being used in clinical practice to manage MTrPs; however, there are no studies supporting any additional beneficial effects over DN. Furthermore, there are cost variations between these techniques, which affect the healthcare system. A cost-effectiveness comparison will determine which treatment intervention is the most efficient.

From a biological point of view, it seems reasonable to hypothesize that subjects can display improvements thanks to the mechanical effects of the needle and that patients may benefit more when the electrolysis effect is added to the mechanical stimulus provided by the needle. Therefore, the aim of this randomized controlled study is to compare the effectiveness of DN versus PNE for reducing the level of pain in patients suffering from PHP.

## Methods

### Sample

The study subjects will be adults of both genders who have been admitted to the Physical Medicine and Rehabilitation Department in a Kuwait City hospital by a medical registered doctor from the Ministry of Health. To be eligible for the study, participants will have to meet the following inclusion criteria:

- Clinical diagnosis of PHP in accordance with the Clinical Guidelines linked to the International Classification of Function, Disability and Health from the Orthopedic Section of the American Physical Therapy Association [3, 4, 16, 18]
- Age between 21 and 60 years at admission to the study, according to the Kuwaiti law
- History of plantar heel pain for over 1 month, showing no improvements with previous conservative treatment
- Able to walk 50 m without any support
- The presence of MTrPs on plantar and calf muscles, based on initial physical examination carried out by a physiotherapist (MA) with experience and training in MTrPs
- Accepting treatment from a male physiotherapist
- The ability to understand the study and the informed consent, as well as having signed the document

Exclusion criteria for the study will be based on:

- Needle phobia
- Needle allergy or hypersensitivity to metals
- Presence of coagulopathy or use of anticoagulants according to medical criteria
- Presence of peripheral arterial vascular disease
- Pregnancy
- Dermatological disease affecting the dry needling area
- The presence of a chronic medical condition which might preclude participation in the study, such as malignancy, systemic inflammatory disorders (e.g., rheumatoid arthritis, psoriatic arthritis, ankylosing spondylitis, septic arthritis), neurological diseases, polyneuropathy, mononeuropathy, and sciatica
- Treatment of plantar heel pain with needling or acupuncture during the last 4 weeks
- A history of injection therapy in the heel over the previous 3 months
- Previous history of foot surgery or fracture

Participants will be controlled by using the appropriate medication dosage as prescribed by the physiatrist (analgesics and non-steroidal anti-inflammatory medications) and will be required to report any changes to the assessor during the evaluations if they take any additional medication or undergo any treatment during the intervention. They must be willing not to receive or implement any form of treatment for the plantar heel pain (taping, night splints, massage therapy, or footwear modifications) while they participate in the trial. The participants will have the right to withdraw from the study at any time without having to provide any explanation.

Regarding sample size, 94 participants with PHP will be recruited. An initial prospective sample size calculation estimated that 39 participants per group will provide 80% power to detect a minimally important difference of 13 points in the pain domain of the Foot Health Status Questionnaire (FHSQ) with a standard deviation of 20 points [23] and an alpha risk at 0.05, allowing 20% loss to follow-up (16 patients).

### Study design

Both the assessment and intervention will take place at the Physical Medicine and Rehabilitation Hospital in Kuwait.

**Fig. 1:**
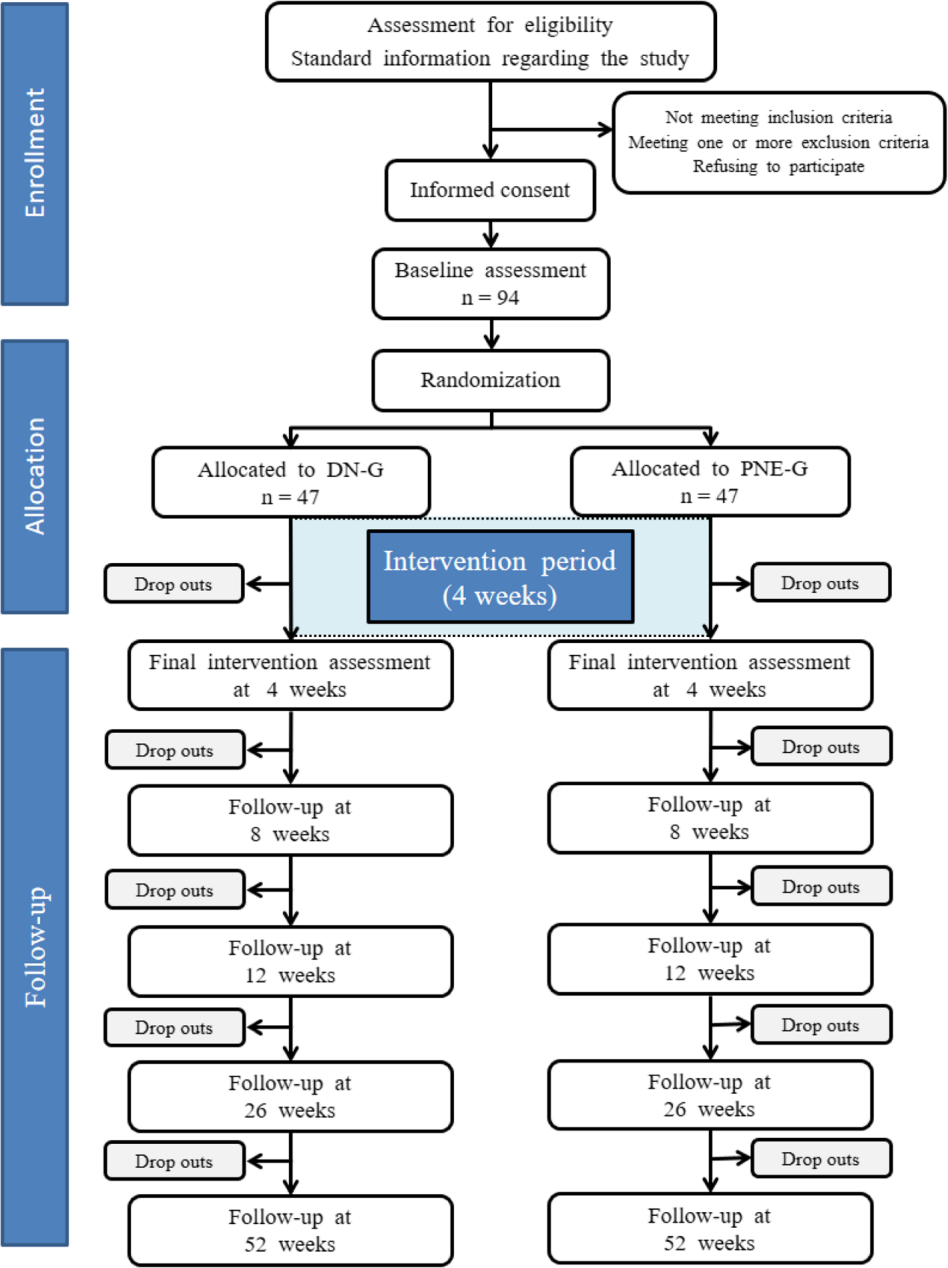
Flow diagram. Randomized controlled trial design

This study is a prospective, two parallel groups (participant) randomized controlled trial with blinded outcome assessment at baseline and at 4, 8, 12, 26, and 52 weeks. The study flow chart shown in Fig. 1 conforms to the Standard Protocol Items: Recommendations for Interventional Trials (SPIRIT) guidelines for nonpharmacological studies [24].

Participants who fulfill the inclusion criteria will receive the standardized oral and written information and, once they consent to participate in the trial, will be randomized in a block system by blocks of 10 patients. Allocation to the groups will be achieved using a computer program (Randomizer, https://www.randomizer.org/) with random patient file number sequences generated by a third person not involved in the study, and based on their file number in Kuwait. This person will be responsible for safekeeping the envelope with the information of the randomization. The envelopes will remain closed until the moment of the intervention in order to maintain the blinding. This professional will also ask the patients for informed consent. This research study was approved by the Medical Ethics Committee of the State of Kuwait Ministry of Health, with reference number 642/2017.

### Interventions

To determine what muscles will be treated, muscles fulfilling the following two criteria will be selected: (a) muscles that typically refer pain to the heel [17] and (b) muscles that can be directly palpated or that can be needled with precision and safety without ultrasound guidance. The clinician will perform a physical examination to find MTrPs following Travell and Simons’ criteria: (1) the presence of a taut band and (2) identification of an exquisite spot tenderness or a nodule [17]. A flat palpation or pincer palpation technique will be used to palpate the MTrPs, depending on the muscle being assessed. The muscles to be treated will be the soleus, gastrocnemius, quadratus plantae, flexor digitorum brevis, and abductor hallucis. If a muscle contains more than one MTrP, the most sensitive MTrP will be treated, according to the patient’s perceived pain upon palpation. If the patient has pain bilaterally, the clinician will treat both sides. The position of the patient will always be lying; however, it depends on each muscle (supine, prone, or lateral decubitus position), and will be the same for the assessment as well as for the intervention [25].

During the first session, all participants will be taught a self-stretching protocol [15] which has demonstrated to be effective for the management of PHP [10, 15, 26] and will consist of the following exercises: (a) Self-stretching of the calf muscles: in standing, with the affected foot furthest away from the wall, the patient will be instructed to lean forward, while keeping the heel on the floor. To focus the stretching on the soleus muscle, the affected knee will be bent, whereas to focus on the gastrocnemius muscle, the affected knee will be kept in full extension. In this position, patients will be taught to lean forward until they feel a stretch in the calf and/or Achilles region. All patients will complete both versions of the stretch. (b) Plantar fascia-specific self-stretching: in the sitting position, patients will cross the affected foot over the contralateral thigh. The patient will place his/her fingers over the base of the toes, grasp the base of the toes, and pull the toes back towards the shin, until a stretch is felt in the plantar fascia [15]. According to the evidence, we will follow the same dosage for calf and plantar fascia-specific self-stretching exercises, twice a day, using intermittent stretching lasting 20 s, followed by 20-s rest periods for a total of 3 min per stretch [15].

Participants will receive four individual physiotherapy sessions, once a week. The duration of the sessions may change depending on the patient; however, these will last approximately 30 min. Participants will be treated by a physical therapist registered at the Kuwait Ministry of Health and trained in the protocol. The clinician will have a minimum of 5 years practical experience in the field of dry needling and appropriate training.

#### Invasive interventional groups: dry needling and percutaneous needle electrolysis

Specific needles for dry needling will be used during invasive treatments (Agu-punt, Spain). Needle length will be determined by the location of the MTrP and will range from 30 to 50 mm in length (or longer if necessary according to patients’ characteristics). The diameter of the needle will be 0.25–0.30 mm. If the participant is sensitive to the needle insertion, the level of manipulation will be reduced. If this measure proves insufficient for reducing the painful stimulus, manipulation of the needle will cease altogether and the needle will be left in situ [25, 27].

To maintain appropriate hygienic conditions during the invasive treatments, the clinician will wear latex gloves and thoroughly clean the skin of the area to be needled with an antiseptic solution (70% Propan-2-ol, Skin-des). Upon removal of the needle, the area will be firmly compressed for 10 s. The needle will be discarded after each single use.

In both groups, the intervention will be terminated in the case of severe adverse effects, if the participant does not wish to continue, and if there is an unapproved use of medication. Any adverse effects will be duly reported.

#### Dry needling arm

Once the clinician locates the MTrP, the needle will be inserted over the same and a rapid needle entry will be performed. The chosen technique for manipulating the needle will be the technique described by Hong [28], which consists of a rapid needle entry and exit (fast in/fast out), in order to obtain a local twitch response (LTR), lasting 5 s employing a rhythmic movement at approximately 1 Hz/sec (five entries). The number of LTRs will be counted and registered.

#### Percutaneous needle electrolysis arm

The electrotherapy equipment used (Physio Invasiva, PRIM Fisioterapia, Spain) produces a continuous galvanic current through the cathode while the patient holds a hand-held anode [22]. Once the needle reaches the relevant treatment area, this will be needled in exactly the same manner as in the DN group, with the only difference being that the needle will be transmitting an electrical current with an intensity of 1.5 mA (intensity may be adapted to patient’s characteristics according to their pain’s tolerance).

### Study variables

#### Baseline data

Baseline data will include gender, age, height, weight, BMI, details regarding the affected side (right, left, or bilateral), duration of symptoms, medication, and previous treatments.

**Fig. 2:**
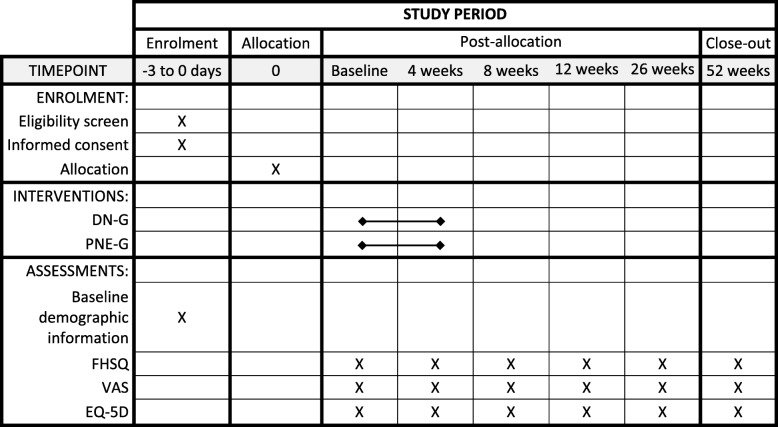
Schedule for enrolment and intervention

A blinded observer will assess all participants at baseline and at 4, 8, 12, 26, and 52 weeks post-treatment (Fig. 2).

#### Primary outcome measure

Participants will complete the FHSQ at baseline and at 4, 8, 12, 26, and 52 weeks post-treatment. The FHSQ consists of 13 questions reflecting four foot health-related domains: pain (4 questions), function (4 questions), footwear (3 questions), and general foot health (2 questions). Individual item scores will then be re-coded, tabulated, and finally transformed to a scale ranging from 0 to 100 for each of the four domains [29]. Greater scores reflect better foot health and quality of life [30]. The FHSQ has been validated [31] and has been used in similar trials that have evaluated the effectiveness of different interventions for plantar heel pain [18, 32, 33].

#### Secondary outcome measures

Participants will complete the visual analogue scale (VAS) at baseline and at the 4-, 8-, 12-, 26-, and 52-week assessments and additionally before each treatment session. The level of pain that patients have experienced during the previous 48 h prior to starting the treatment session will be recorded. Participants will be asked about the mean and the highest level of pain they have experienced. The exact wording of the questions will be: (1) what is the level of pain, on average, that you have felt during the last 48 h? and (2) what is the maximum level of pain you have felt during the last 48 h? Additionally, after treatment, they will be asked to score their current pain immediately upon standing up and walking a few steps. Participants will be explained that a score of 0 indicates the absence of pain, whereas a score of 10 represents the maximum tolerable pain. The VAS is widely used and is valid and reliable [34–36]. They will also indicate the areas of perceived pain on an electronic body chart (Navigate pain, version 0.1.9.9, Aalborg, Denmark) [37].

Quality of life (QoL) will be assessed with the EuroQoL-5 dimensions (EQ-5D), which will be filled out by the patients at baseline and at the 4-, 8-, 12-, 26-, and 52-week assessments. The EQ-5D self-report questionnaire is a descriptive system with five questions, each representing one dimension of health-related quality of life (HRQoL), i.e., mobility, self-care, daily activities, pain/discomfort, and depression/anxiety. Each dimension can be rated on three levels: no problems, some problems, and major problems, and together, the results serve to classify people into 1 of 243 possible health states [38].

#### Cost analysis

Costs will be collected from the healthcare viewpoint. Direct healthcare costs are the costs of manual therapy, physiotherapy or general practitioner care, additional visits to other healthcare providers, drugs, and hospitalization. Cost-effectiveness and cost-utility analyses will be carried out with quality-adjusted life-year, estimated from EQ-5D scores.

### Statistical analysis

The statistical analysis will be performed via an intention-to-treat analysis. Variables will be described in number (percentage) and average (standard deviation) or median (interquartile range), according to their distribution. Quantitative variables will be analyzed with the Shapiro Wilk test in order to confirm their distribution and to determine the correct statistical tests according to these results.

The outcomes will be analyzed using mixed linear and logistic regression models considering participants as a random effect and treatment group as fixed factors.

Baseline characteristics will be introduced in the model as covariance factors. The numbers needed to treat index will also be calculated. The primary aim of the analysis will be to calculate the difference obtained in the FHSQ score after the intervention (final measurement − initial measurement). Finally, the magnitude of the effect of the result will be calculated and, therefore, its clinical importance, by using the following formula: *r* = √[***F(1,dfR)]/[F(1,dfR)+dfR***].

The significance level for statistical tests will be set at *p* ≤ 0.05.

### Ethics and dissemination

The study design, procedures, and informed consent procedure were approved by the Ministry of Health in the state of Kuwait on 19 September 2017, and the study will be conducted in compliance with the Helsinki Declaration of Human Rights. The registration number provided by ClinicalTrials.gov is NCT03236779 (registered 2 August 2017). Participants will be requested to provide informed written consent before randomization. The software used to assemble the papers included in this review will be EndNote X7 v17.0.1. The participant data obtained in this ongoing research will not to be used for other purposes. All the personal information collected such as the informed consent form and the physical examination findings will be stored by category in a specific filing cabinet before, during, and after the trial, in order to protect confidentiality. After completing the data analysis, and regardless of the findings, we plan to disseminate all the trial results via conferences and publications.

## Discussion

Plantar heel pain is a common cause of foot pain and discomfort affecting the health and quality of life of patients, with a high tendency for relapse and chronicity [8]. Previous studies have demonstrated the positive effect of conservative treatment in reducing painful conditions associated to PHP [15, 19], while other RCTs show that DN probably has a higher potential benefit over more conservative approaches [16]. Nevertheless, according to systematic reviews, new high-quality RCTs are needed on which to base the evidence regarding the effectiveness of DN for symptoms management in PHP [39]. Despite the fact that the plantar fascia can be a source of pain in itself [40] and that other studies performing invasive treatments have considered needling upon the insertion of the plantar fascia [41], our hypothesis is restricted to evaluating the contribution of MTrPs towards PHP.

As an innovative treatment modality, PNE is being increasingly used in order to promote the regeneration of injured tendons [20–22, 42] and is being gradually recognized as a cornerstone for invasive approaches in physiotherapy. However, despite the fact that its use is increasing based on an apparently additional effect to only DN, there is no scientific evidence to support the use of this technique in clinical practice. Due to this fact, our aim is to research whether PNE can offer an additional effect to DN for PHP management. To our knowledge, this will be the first study to compare two invasive treatments for MTrPs associated with PHP. Not only this study will contribute to research regarding the possible additional effects of PNE, but also by analyzing differences in pain perception after therapy, it will address a common patient complaint. Furthermore, cost-effectiveness data will be extracted based on the EQ-5D, thus providing a valuable economic variable to studies involving physiotherapy techniques.

## Abbreviations

BMI: Body mass index
DN: Dry needling
DN-G: Dry needling group
EQ-5D: EuroQol-5D
FHSQ: Foot Health Status Questionnaire
HRQoL: Health-related quality of life
LTR: Local twitch response
MTrP: Myofascial trigger point
PHP: Plantar heel pain
PNE: Percutaneous needle electrolysis
PNE-G: Percutaneous needle electrolysis group
QoL: Quality of life
RCT: Randomized clinical trials
ROM: Range of motion
SPIRIT: Standard Protocol Items: Recommendations for Interventional Trials
SSHP: Self-stretching home program
VAS: Visual analogue scale
